# Epigenetically silenced GNG4 inhibits SDF1α/CXCR4 signaling in mesenchymal glioblastoma

**DOI:** 10.18632/genesandcancer.105

**Published:** 2016-03

**Authors:** Jagriti Pal, Vikas Patil, Baisakhi Mondal, Sudhanshu Shukla, Alangar S. Hegde, Arimappamagan Arivazhagan, Vani Santosh, Kumaravel Somasundaram

**Affiliations:** ^1^ Department of Microbiology and Cell Biology, Indian Institute of Science, Bangalore, India; ^2^ Sri Satya Sai Institute of Higher Medical Sciences, Bangalore, India; ^3^ Departments of Neuropathology and Neurosurgery, National Institute of Mental Health and Neuro Sciences, Bangalore, India

**Keywords:** glioblastoma, DNA hyper methylation, G-protein, G-protein-coupled receptors, guanine nucleotide binding-protein gamma subunit 4, chemokine receptor, CXCR4, SDF1α

## Abstract

The most common and aggressive form of primary brain tumor in adults is glioblastoma (GBM). From the global DNA methylation profiling study, previously published from our laboratory, we identified *G*uanine *N*ucleotide binding-protein *G*amma subunit *4* (GNG4) to be one of the most hyper methylated and down regulated genes in GBM. GBM derived cell lines showed reduced GNG4 transcript levels, which could be reversed by methylation inhibitor treatment. Bisulphite sequencing confirmed the methylation status in glioblastoma tumor tissue and GBM derived cell lines. Overexpression of GNG4 was found to inhibit proliferation and colony formation of GBM cell lines and *in vitro* transformation of immortalized human astrocytes, thus suggesting a potential tumor suppressor role of GNG4 in GBM. Correlation of GNG4 transcript levels with that of all GPCRs from TCGA data revealed chemokine receptors as the potential target of GNG4. Furthermore, exogenous over expression of GNG4 inhibited SDF1α/CXCR4-dependent chemokine signaling as seen by reduced pERK and pJNK and GBM cell migration. The inhibitory association between GNG4 and SDF1α/CXCR4 was more evident in mesenchymal subtype of GBM. Thus, this study identifies GNG4 as an inhibitor of SDF1α/CXCR4-dependent signaling and emphasizes the significance of epigenetic inactivation of GNG4 in glioblastoma, especially in mesenchymal subtype.

## INTRODUCTION

Grade IV astrocytoma or glioblastoma (GBM) is the most common and aggressive form of brain tumor in adults. With the current treatment modality which includes surgery, radiotherapy and temozolomide chemotherapy, the overall median survival achieved till now is only 14.6 months [1, 2]. During tumor development, cells accumulate numerous genetic and epigenetic changes to acquire the characteristics of proliferation, survival, invasion and angiogenesis [3].

Epigenetic mechanisms play an important role in normal development and disease conditions [4]. There are many epigenetic mechanisms that can cause dynamic alterations in the transcriptional profile of cells, of which DNA methylation plays a major role in the etiology of common human diseases like cancer, multiple sclerosis, schizophrenia etc. [5, 6]. Hyper methylation of the promoter region of tumor suppressor genes have been firmly established as a mechanism for oncogenesis [7]. In the mammalian cell, DNA methylation occurs in the C5 position of CpG di-nucleotides and is carried out by a class of enzymes known as the DNA methyltransferases. DNA methylation leads to altered gene expression either through recruitment of proteins involved in gene repression or through inhibition of binding of transcription factors to the DNA [8].

G-Protein Coupled Receptors (GPCRs) constitute a large family of receptors that respond to various extracellular stimuli like hormone, growth factor, sensory stimulating signals like light etc. Signaling via GPCRs can modulate various pathways like MAPK, PI3K and RhoGEF pathways, and also alter levels of secondary messengers like cAMP and Ca^2+^. G-protein trimers, comprising of α, β and γ subunits, are responsible for mediating signals from GPCRs to the inside of the cell. The α subunit generally activates effector molecules post GPCR activation while the βγ heterodimer behaves as regulators of the signal [9, 10]. Analysis of global DNA methylation profiling of GBM samples using Illumina Infinium 27K methylation array, previously published from our laboratory [11], revealed Guanine Nucleotide binding protein γ subunit 4 (GNG4) to be one of the most hyper methylated and down regulated genes in GBM patients. GNG4 is one of the fourteen γ subunits of the human genome [12]. In the current study, we try to understand the role of GNG4 as a tumor-suppressor in GBM and also elucidate the GPCR signaling which is regulated by it.

## RESULTS

### GNG4 is hyper methylated and down regulated in GBM

**Figure 1:**
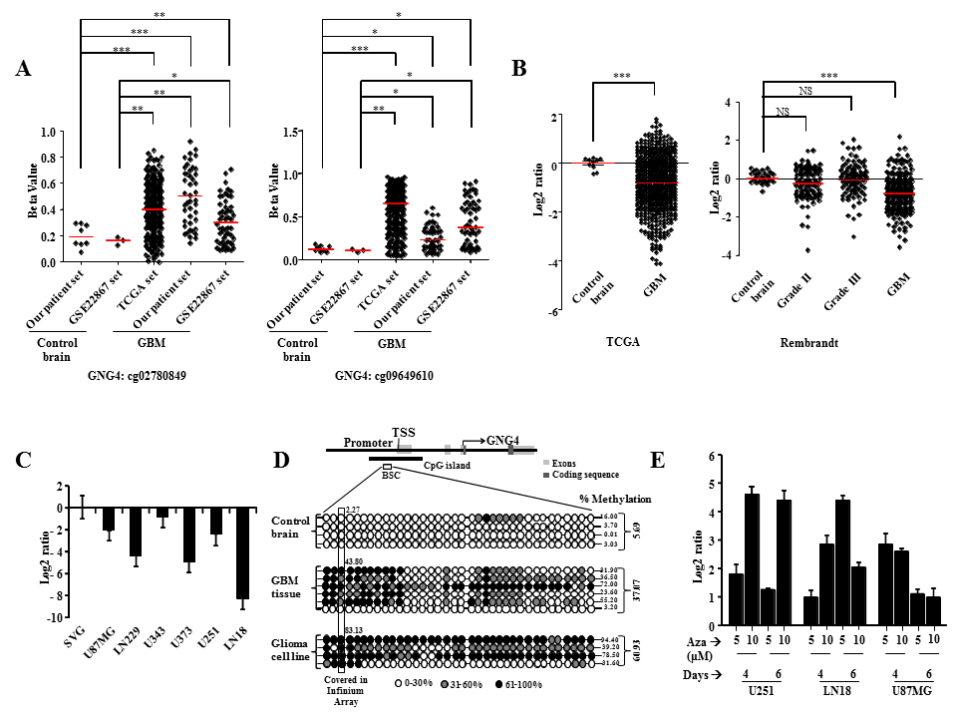
GNG4 is hyper methylated and down regulated in GBM A) Methylation levels of two CpGs from 27K methylation array (Illumina) present in the promoter region of GNG4 in three datasets – our patient set, GSE22867 and TCGA, B) RNA level expression of GNG4 in TCGA and Rembrandt datasets, C) RNA level of GNG4 in GBM cell lines as compared to immortalized normal human astrocyte cell line, SVG, D) Bisulphite sequencing of the promoter region of GNG4 encompassing 36 CpG islands in control brain, GBM tissue and GBM cell lines. The percentage of methylation in all CpGs of the GNG4 promoter sequence has been given at the right side of each sample. The average percentage methylation of the CpG covered in Illumina Infinium Array has been provided near the box corresponding to that CpG. BSC refers to the bisulphite converted region that is subjected to sequencing, E) RNA level of GNG4 after treatment of GBM cell lines with DNA methyltransferase inhibitor, Azacytidine (Aza). p-value was calculated by Student's t test where *, ** and *** represents p-value of < 0.05, < 0.01 and < 0.001 respectively.

In a previous study, we carried out genome-wide DNA methylation analysis of GBM patients using Illumina 27K methylation array [11]. Hyper methylated genes were analyzed for their gene expression status from TCGA microarray data to find out genes which are hyper methylated as well as down regulated as compared to control brain samples [11]. From this, we identified GNG4 to be one of the most hyper methylated and down regulated genes in GBM. The methylation levels of the two CpG probes from Illumina 27K methylation array (i.e. cg02780849 and cg09649610), both present in the GNG4 promoter region, were checked in TCGA, our patient set and GSE22867 datasets (Figure 1A). Both the CpGs were found to be significantly hyper methylated in GBM samples of all three datasets compared to control brain samples of our patient set (Figure 1A). Additionally, both the CpGs were found to be significantly hyper methylated in GBM samples of all three datasets compared to control brain samples of GSE22867 dataset (Figure 1A). Promoter hyper methylation leads to transcriptional repression [13]. As expected, the GNG4 transcript level was found to be significantly down regulated in GBM compared to control brain tissue in TCGA and Rembrandt datasets (Figure 1B). Consequently, we also observed a reduced level of GNG4 RNA in GBM cell lines as compared to immortalized normal human astrocyte SVG (Figure 1C). To confirm promoter hyper methylation, bisulphite sequencing of the CpG island located in the GNG4 promoter region was carried out which revealed an average of 37% methylation in GBM tissue samples and 61% methylation in GBM cell lines compared to only 6% methylation in control brain samples (Figure 1D). To validate whether down regulation of GNG4 transcript levels is indeed a direct effect of promoter methylation, methylation inhibitor (azacytidine) treatment was carried out in GBM cell lines and this indeed resulted in the re-expression of GNG4 transcript to varying levels (Figure 1E). From these results, we conclude that the promoter region of GNG4 is hyper methylated in GBM as compared to control brain tissue and this ultimately leads to reduced expression of GNG4 in GBM.

### GNG4 inhibits the growth of GBM cells

**Figure 2:**
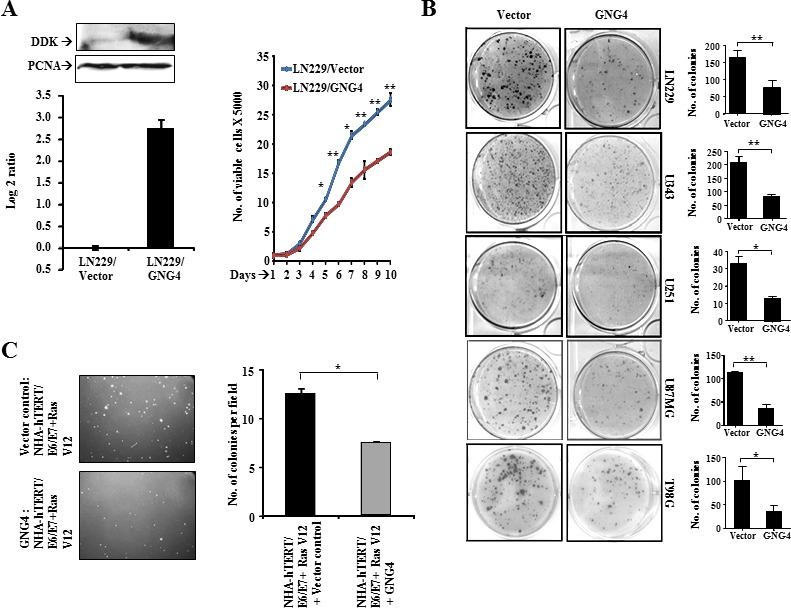
Effect of GNG4 ectopic overexpression in GBM cell lines A) Overexpression of GNG4 in LN229 cells – top: protein level, bottom: RNA level, right: Number of proliferating (viable) cells for LN229/Vector and LN229/GNG4 conditions measured every 24 hours for ten days, B) Colony formation capacity of GBM cells, C) Role of GNG4 in inhibition of transformation of immortalized normal human astrocytes (NHA-hTERT/E6/E7) by RAS V12 oncogene. p-value was calculated by Student's t test where *, ** and *** represents p-value of < 0.05, < 0.01 and < 0.001 respectively.

GNG4 being silenced by promoter methylation in GBM, we hypothesized that its expression might be growth inhibitory to cells. GNG4 was ectopically over expressed in GBM cell lines and its tumor suppressor functions were tested (Figure 2). Ectopic over expression of GNG4 indeed resulted in several fold increase in GNG4 transcript and protein levels in LN229 cells (Figure 2A). Further, proliferation of LN229 cells stably expressing GNG4 (LN229/GNG4) was found to be significantly lower compared to that of vector control cells (LN229/Vector) (Figure 2A). Colony formation assay, which measures the growth of cells for longer period of time, revealed that GNG4 stable GBM cells forms significantly fewer colonies than vector stable GBM cells (Figure 2B). GNG4, a γ subunit of the G protein complex, has the potential to negatively regulate signaling downstream of oncogenic GPCRs. Activated GPCRs have been associated with transformation [14-17]. Since GNG4 over expression resulted in growth inhibition, we conjectured that GNG4 expression might inhibit transformation. We tested the effect of ectopic introduction of GNG4 on the ability of RasV12 mutant protein to transform immortalized (E6/E7 and hTERT) astrocytes. We found that Ras-induced transformation was inhibited significantly by GNG4 (Figure 2C, compare grey bar with black bar). From the above data, we conclude that GNG4 indeed is a potential tumor suppressor in GBM.

### GNG4 targets chemokine-chemokine receptor signaling pathway

**Figure 3:**
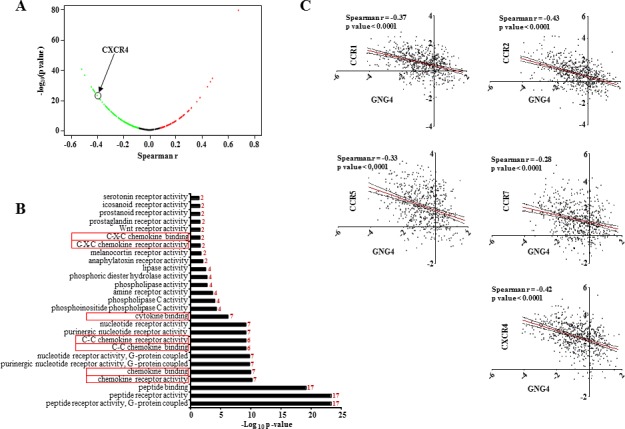
CXCR4 is a potential GPCR regulated by GNG4 A) Volcano plot of correlation of RNA levels of GNG4 with that of all GPCRs in GBM samples of TCGA (n=572), CXCR4 is marked with black circle, B) Gene Ontology pathway enrichment analysis of GPCRs negatively correlating with GNG4. The number to the right side of each bar represents the number of genes enriched. Please note that, chemokine-chemokine receptor signaling got enriched multiple times (denoted by red box), C) Correlation of RNA level of GNG4 with that chemokine receptors CCR1, CCR2, CCR5, CCR7 and CXCR4 in all GBM samples in TCGA (n=572).

There are over 800 GPCRs reported in mammalian cells and many of them have been shown to be activated in cancer cells [18]. GNG4, being a γ subunit of the G protein trimer, potentially functions as a negative regulator of GPCR signaling. Since GNG4 is a growth inhibitory protein that is down regulated in GBM, we hypothesized that GNG4 should be regulating an oncogenic GPCR in GBM. Many GPCRs including chemokine receptors, lysophosphatidic acid receptors etc. get activated in cancer by over expression [19, 20]. To find out a potential GPCR regulated by GNG4, first we correlated the GNG4 transcript levels with that of 320 GPCRs for which transcriptome data was available from TCGA dataset. We particularly looked at GPCRs whose expression level was negatively correlating with that of GNG4 because we conjectured that GNG4 should be down regulated in the scenario where an oncogenic GPCR will be over expressed. The search identified that several GPCRs have significant negative correlation with GNG4 transcript levels (Figure 3A and Supplementary Table ST1). Further, an unbiased functional enrichment analysis was carried out using above negatively correlating GPCRs to identify pathways regulated by GNG4 (Supplementary Table ST1). Under molecular functions category of gene ontology analysis, several important terms got enriched. While several GPCRs related to peptide receptor activity were enriched, chemokine-chemokine receptor interaction pathway was particularly striking as it was enriched several times (Figure 3B and Supplementary Table ST2). Moreover, chemokine-chemokine receptor interaction has been implicated in cancer development and progression including GBM [21]. Additional investigation revealed that seven out of sixteen chemokine receptors (CCR1, CCR2, CCR5, CCR7, CCRL1, CXCR4 and CXCR7) are up regulated in GBM (Supplementary figure 1A and 1B). More specifically, we found C-C family chemokine receptors-CCR1, CCR2, CCR5 and CCR7 and C-X-C family receptor-CXCR4 to be up regulated in GBM and also have a significant negative correlation with GNG4 transcript levels (Figure 3C). Thus we conclude from this section that chemokine receptors -CCR1, CCR2, CCR5, CCR7 and CXCR4 could be potential targets of GNG4 in GBM.

### C-X-C family chemokine receptor CXCR4-dependent signaling is inhibited by GNG4

**Figure 4:**
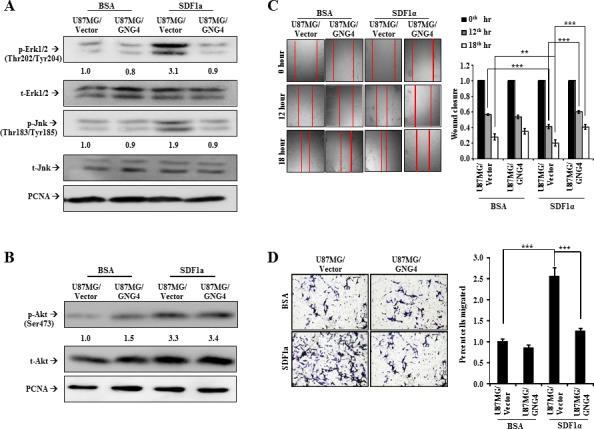
Effect of GNG4 on CXCR4/SDF1α signaling and GBM cell migration A) Regulation of CXCR4 signaling by GNG4 tested by measuring the levels of phospho-Erk1/2 and phospho-Jnk, B) Regulation of CXCR4 downstream AKT pathway by GNG4 tested by measuring the levels of phosphorylation of Ser473 of Akt, C) Scratch assay in U87MG cell lines where CXCR4 signaling was activated using exogenous purified SDF1α and simultaneously GNG4 was overexpressed. Right: Quantification of scratch assay, D) Migration of U87MG cells tested using Boyden chamber assay. CXCR4 was activated using purified SDF1α. In the same condition, GNG4 was ectopically overexpressed to understand the effect of GNG4 in CXCR4 mediated migration of GBM cell lines. Right: Quantification of Boyden chamber assay. p-value was calculated by Student's t test where *, ** and *** represents p-value of < 0.05, < 0.01 and < 0.001 respectively.

Among the chemokine receptors identified as potential targets of GNG4, we chose to study CXCR4 for the following reasons. The chemokine receptor CXCR4 is one of the top GPCRs negatively correlating with GNG4, it is highly expressed in GBM [22] (Supplementary figure 1) and it plays a major role in GBM cell proliferation, migration and invasion [23, 24]. Activation of CXCR4 by its ligand SDF1α leads to activation of AKT, JNK and MAPK pathways [23, 25]. CXCR4 has been shown to interact with Gα subunit resulting in the activation of ERK signaling and JNK signaling pathways, both of which play important roles in cell migration [26, 27]. To evaluate the effect of CXCR4 activation and subsequent regulation by GNG4 in GBM context, we carried out experiments in U87MG cell line because these cells express detectable amount of the receptor [23]. We found that the addition of SDF1α to U87MG vector stable cells (U87MG/Vector) increases phospho-Erk1/2 and phospho-Jnk levels (Figure 4A). However, SDF1α addition to U87MG cells stably over expressing GNG4 (U87MG/GNG4) failed to show increase in phospho-Erk1/2 and phospho-Jnk levels (Figure 4A). We also looked at the effect of GNG4 on AKT pathway, by measuring phosphorylation of Serine 473 of Akt, which revealed that although SDF1α could induce activation of Akt, over expression of GNG4 in that condition failed to inhibit Akt phosphorylation (Figure 4B). In fact, in control BSA treated condition; there was an increase in Akt phosphorylation when GNG4 was over expressed (Figure 4B). This could be attributed to the fact that Gβγ subunits by themselves can activate various downstream effector molecules including PLCβ, which leads to activation of Akt [28]. CXCR4 receptor dependent activation of ERK and JNK signaling pathways has been shown to increase cell migration [26]. Hence, the role of GNG4 in CXCR4 mediated GBM cell migration was evaluated. Scratch assay was performed in U87MG cell line in presence or absence of SDF1α with simultaneous over expression of GNG4. The wound closure was measured 12 hours and 18 hours after the scratch was made. It was observed that, at both 12^th^ hour and 18^th^ hour, activation of CXCR4 signaling by SDF1α increased the migration capacity of U87MG/Vector stable cells which gets severely abrogated in U87MG/GNG4 stable cells (Figure 4C). Cell migration capacity measured by Boyden chamber assay also showed that SDF1α is able to induce migration efficiently in U87MG/Vector stable cells, but not in U87MG/GNG4 stable cells (Figure 4D).

**Figure 5:**
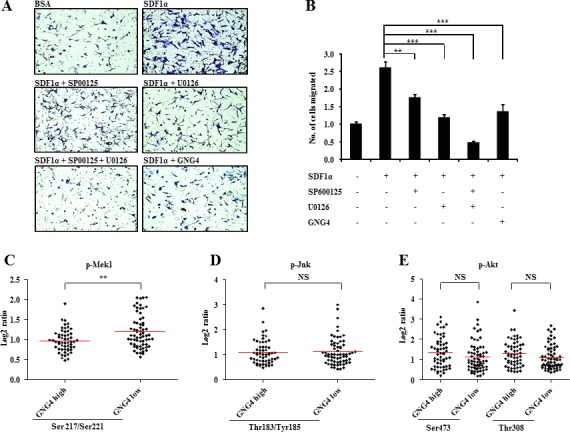
Importance of CXCR4 downstream pathways in regulation of GBM cell migration A) Role of CXCR4 downstream pathways, ERK and JNK pathways, in GBM cell migration. ERK and JNK pathways were inhibited by respective inhibitors and migration of U87MG cells were tested in Boyden chamber, B) Quantification for the same experiment, C) Levels of phospho-Mek1 in CXCR4 high-GNG4 high and CXCR4 high-GNG4 low conditions, D) Levels of phospho-Jnk in CXCR4 high-GNG4 high and CXCR4 high-GNG4 low conditions, E) Levels of phospho-Akt (Ser473 and Thr308) in CXCR4 high-GNG4 high and CXCR4 high-GNG4 low conditions. p-value was calculated by Student's t test where *, ** and *** represents p-value of < 0.05, < 0.01 and < 0.001 respectively.

Our next objective was to find out the importance of ERK and JNK pathways in SDF1α/CXCR4 mediated cell migration. We inhibited each of these two pathways using pharmacological inhibitors and measured the cell migration in SDF1α treated conditions. It was observed that, treatment with inhibitors for both Jnk (SP600125) and Erk (U0126) led to significant inhibition of the migration capacity of U87MG GBM cell line, although the effect was more pronounced when Erk was inhibited as compared to the inhibition of Jnk (Figure 5A and 5B). Furthermore, simultaneous inhibition of both Erk and Jnk in CXCR4-activated condition led to almost five fold reduction in migration capacity. Activation of GPCRs by their ligands will be dependent on downstream signal modulators. Hence, in this context, presence of SDF1α may not elicit CXCR4 signaling if the downstream regulator, GNG4, is not down regulated. To validate this hypothesis, we looked at the levels of phosphorylation of downstream molecules of ERK, JNK and AKT pathways in GNG4 high and GNG4 low conditions from TCGA data. We selected samples where CXCR4 RNA levels are high (Log2 ratio > 2) i.e., tumor cells which might be dependent on CXCR4 signaling, and divided those patients into GNG4 high (RNA Log2 ratio > −0.79) and GNG4 low (RNA Log2 ratio < −0.79) groups. In these two groups, we checked the levels of phosphorylation of Mek1, Jnk and Akt from TCGA Reverse Phase Protein Array (RPPA) data (Figure 5C, 5D and 5E respectively). We observed that phospho-Mek1 levels are significantly higher in GNG4 low group as compared to GNG4 high group (Figure 5C). This was not observed in case of phospho-Jnk or phospho-Akt (Figure 5D and 5E). This could be because ERK pathway gets highly activated when CXCR4 signaling is triggered (Figure 4A) which is reinforced by the fact that inhibition of ERK pathway leads to considerably higher attenuation of GBM cell migration as compared to when JNK pathway is inhibited (Figure 5A). From all these results, it is evident that GNG4 inhibits SDF1α/CXCR4 signaling mediated GBM cell migration through abrogation of mainly the ERK signaling pathway.

### Down regulation of GNG4 is essential for activation of CXCR4 signaling in mesenchymal GBM subtype

**Figure 6:**
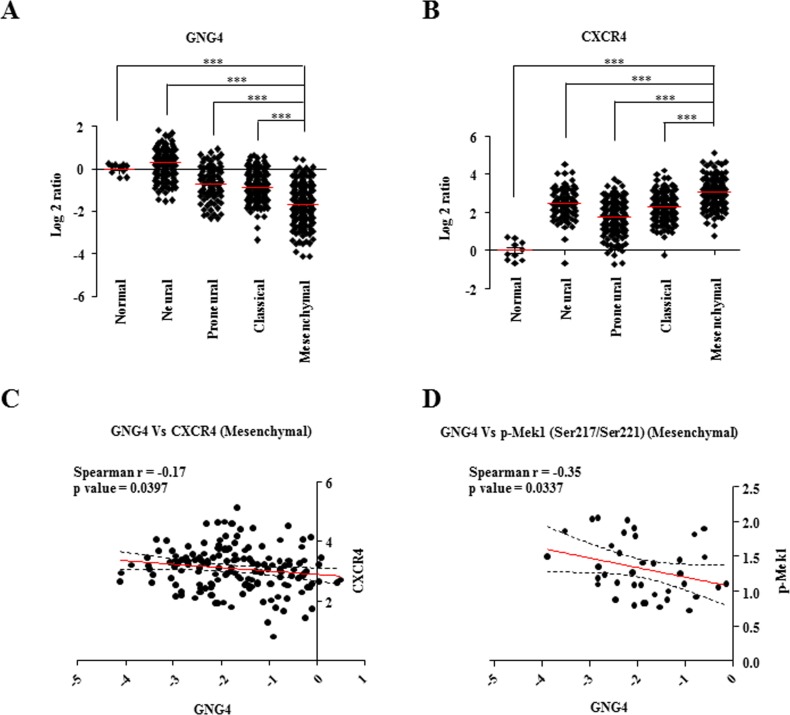
Expression of GNG4 and CXCR4 in different GBM subtypes A) RNA level of GNG4 in different GBM subtypes, B) RNA level of CXCR4 in different GBM subtypes, C) Correlation of RNA level of GNG4 with that of CXCR4, D) Correlation of RNA level of GNG4 with protein levels of ERK upstream molecule, phospho-Mek1. p-value was calculated by Student's t test where *, ** and *** represents p-value of < 0.05, < 0.01 and < 0.001 respectively.

Based on certain genetic and epigenetic alterations, GBM is classified into four molecular subtypes-neural, proneural, classical and mesenchymal [29]. Mesenchymal subtype of GBM is characterized by increased malignancy and infiltrative nature compared to the other three subtypes [29-31]. To dissect the importance of the inhibitory effect of GNG4 on CXCR4-dependent signaling and cell migration in different subtypes, we assessed the expression pattern and the correlation of GNG4 and CXCR4 within the subtypes of GBM from TCGA data. It was observed that the RNA level of GNG4 is least and that of CXCR4 is highest in mesenchymal GBM subtype (Figure 6A and B). Additionally, significant negative correlation between GNG4 and CXCR4 transcripts was seen only in mesenchymal subtype (Figure 6C, Supplementary figure 2A). We next checked the correlation between GNG4 transcripts and phospho-Mek1, phospho-Jnk and phospho-Akt (Ser473 and Thr308) levels scored by RPPA data in TCGA in different GBM subtypes. A significant negative correlation was observed between GNG4 and phospho-Mek1 (Spearman r = −0.35) only in mesenchymal subtype (Figure 6D; Supplementary figure 2B). However, GNG4 RNA and phospho-Jnk or phospho-Akt levels did not correlate in any of the subtypes (Supplementary figure 3A, 3B and 3C). Collectively from all these results, we conclude that epigenetic silencing of GNG4 in glioblastoma, specifically the mesenchymal subtype, is essential because its expression would inhibit GBM cell migration mainly through inhibition of ERK pathway downstream to SDF1α/CXCR4-dependent signaling.

## DISCUSSION

GPCRs represent the single largest class of membrane proteins in the human genome. There are over 800 unique GPCRs, of which approximately 460 were predicted to be olfactory receptors [32]. The natural ligands of GPCRs range from subatomic particles (a photon), to ions (H^+^ and Ca^++^), to small organic molecules, to peptides and proteins [33]. Twenty-five years ago, the first GPCR, rhodopsin, was identified [14]. Subsequently, various other GPCRs including β-adrenergic receptors [34, 35], adenosine receptors [36], dopamine receptors [37], chemokine receptors [38] etc. were identified. Signaling via GPCRs can activate various pathways including pro-urvival and pro-proliferative pathways like MAPK, PI3K, JNK and β-Catenin pathways [39]. Chemokine receptors, originally known to function as activators of immune cells, can activate such pro-proliferative pathways and have been shown to have huge implications in tumor cell growth [40]. Various chemokine receptors are highly up regulated in different types of cancers and play important roles in tumor growth, metastasis and angiogenesis [40]. In most cases of signaling through GPCRs, G-protein trimers are responsible for mediating signals from the GPCRs to the inside of the cell where the Gα subunits carry the signal from GPCR while Gβγ dimer regulates the same [10]. In the current study, we elucidate the role of a particular γ subunit of the G-protein trimer as a tumor suppressor in GBM through regulation of a chemokine receptor.

GNG4 is one of the fourteen γ subunit proteins of the G-protein trimer complex [12] and we have shown previously that it is hyper methylated and down regulated in GBM [11]. The importance of GNG4 as a potential tumor suppressor was evaluated by ectopically over expressing the gene in GBM cell lines. It was observed that GNG4 over expression leads to a significant abrogation in proliferation of GBM cell lines as well as transformation of immortalized normal human astrocytes by RAS V12 oncogene. GNG4 as a potential tumor suppressor has been previously shown in renal cell carcinoma [41]. Here, we provide evidence that GNG4 behaves as a tumor suppressor in GBM scenario.

Integrative analysis was carried out to evaluate potential GPCRs that may be regulated by GNG4 in GBM and this revealed chemokine receptors to be potential oncogenic GPCRs regulated by GNG4. Chemokine receptor CXCR4 is very highly up regulated in GBM and it is known to play important role in GBM cell proliferation and migration [23, 24]. In this study, we establish that CXCR4 signaling is regulated by GNG4 because over expression of GNG4 in CXCR4 activated condition failed to induce phosphorylation of CXCR4 downstream signaling molecules, Erk1/2 and Jnk. The impedance of the signaling axis through CXCR4 by GNG4 was able to inhibit migration of GBM cells.

CXCR4 is highly expressed in 23 different cancers of various origins including GBM [23, 24, 42]. However, activation of GPCRs by their ligands may not be sufficient to drive the signaling through them if the downstream molecules fail to be modulated accordingly [43]. Here, we show that CXCR4 downstream molecule, G-protein γ subunit GNG4, is required to be down regulated in GBM for activation of ERK and JNK pathways that ultimately lead to GBM cell migration. Moreover, in GBM patients from TCGA data, we observe that phospho-Mek1, and not phospho-Jnk, levels are higher in GNG4 low tumor as compared to those where GNG4 is high, which is corroborated by the fact that chemical inhibitor against ERK pathway abrogates GBM cell migration to the maximum extent. Although in cell line-based experiments, GNG4 abrogated both phospho-Erk and phospho-Jnk, but it is evident that in tumor scenario ERK pathway has a more pronounced role and hence is modulated to a greater extent by GNG4.

Since GNG4 was found to be down regulated in GBM and not in grade II and grade III astrocytomas, it is evident that GNG4 has important role to play in GBM aggressiveness. Indeed, when we looked at the gene expression levels of GNG4 and CXCR4 in different GBM subtypes, GNG4 was most down regulated and CXCR4 was most up regulated in mesenchymal subtype of GBM which is characterized by more malignant and invasive phenotype as compared to classical, neural and proneural [30, 31]. Additionally, only phospho-Mek1 correlated negatively with GNG4 in mesenchymal subtype while phospho-JNK and phospho-Akt did not. From this study, it is evident that GNG4 is a tumor suppressor in GBM which functions by abrogating the migration property of GBM cells through inhibition of mainly the ERK pathway downstream to CXCR4/SDF1α signaling axis. These results confirm the necessity of epigenetic silencing of GNG4 in GBM, specifically in mesenchymal subtype.

Collectively, we conclude that in a subset of GBM patients, down regulation of GNG4 plays a major role in activation of CXCR4 pathway. Here we see that mesenchymal subtype of GBM, characterized by highly infiltrative tumor [31], has very high levels of CXCR4 and very low levels of GNG4 compared to the other subtypes which suggests that CXCR4 pathway might be a primary oncogenic signaling pathway in this type of tumor. Hence, targeting CXCR4 pathway in combination with existing therapies is more likely to succeed in mesenchymal GBM. This could be achieved by targeting the GPCR itself by using small molecule antagonist like AMD3100 which is proved to be potentially effective therapy in multiple cancers [44-47]. Hence, from this study it can be inferred that therapy using CXCR4 inhibitor should be concentrated on mesenchymal GBM patients who have tumor cells dependent on CXCR4 axis. Hence, it is important to know the status of both CXCR4 up regulation and GNG4 down regulation for the inhibitors to act effectively in reducing tumor cell migration and infiltration.

## MATERIALS AND METHODS

### Cell lines and plasmid constructs

The GBM cell lines U87MG, LN229, U373, U343, U251 and LN18 and the normal human astrocytes SVG [48] and NHA-hTERT-E6/E7 [49] were cultured in Dulbecco's Modified Eagle's Medium (DMEM) and 10% FBS at 37oC and 5% CO. U343, LN18, NHA-hTERT-E6/E7 and SVG were obtained from the laboratory of Dr. A. Guha, University of Toronto, Canada. U87MG, T98G, U251, LN229 and U373 were obtained from Sigma Aldrich (Saint Louis, Missouri, USA). Over expression construct of DDK-tagged GNG4 was purchased from Origene along with corresponding vector control pCMV-Entry. RAS V12 over expression construct was a kind gift from Dr. Annapoorni Rangarajan (IISc).

### Proliferation assay

LN229 cells stably expressing vector control (VC) or GNG4 were plated (5000 cells) in 12-well dishes in duplicates. Proliferation was measured in each day at 24 hours interval. Cells were harvested and stained with trypan-blue and viable cells were counted using hemocytometer in triplicates.

### Colony suppression assay

GBM cells (LN229, U343, U251, U87MG and T98G) were transfected with VC or GNG4 vectors and selected in 500-1000 ug/ml of G418 for two weeks to select stably expressing cells. For each cell line, VC and GNG4 expressing cells were plated in 6-well plate (5000 cells/well) and allowed to grow for two weeks under selection of G418. Colonies were quantified at the end of experiment.

### Migration assay

1.5 × 10^5^ cells were plated in 12 well dishes. Duplicates were plated for each of VC and GNG4 expressing cells. After 24 hours, a scratch was made using a small tip and cells were allowed to migrate in serum-free DMEM. Pictures were taken at 0^th^, 12^th^ and 18^th^ hour and distance of cells migrated was quantified using ImageJ software.

### Transformation assay

0.35 × 10^6^ NHA-hTERT-E6/E7 cells were plated in each of 35 mm dishes. Cells were co-transfected with RAS V12 overexpressing construct along with either VC or GNG4 construct. After 24 hours, 10,000 cells were plated in soft-agar for transformant cells to form colonies.

### Western blotting

Cells were harvested and lysed using RIPA buffer. 300 ug of protein was loaded in each well and SDS-PAGE was carried out. The antibodies used were DDK (TA-50011, Origene), phospho-Erk1/2 (#9101, CST), total-Erk (#9102, CST), phospho-Jnk (#9251 CST), total-Jnk (#9252, CST), phospho-Akt (#9271, CST), total-Akt (#4691, CST) and PCNA (NA-03, Calbiochem).

### Real-time qPCR

Total RNA was isolated by harvesting cells in Trizol reagent (Sigma) followed by chloroform-isopropanol method. For cDNA conversion, 2 μg good quality RNA was used per reaction. Applied Biosystems™ High Capacity cDNA Reverse Transcription kit (Part no. 4368813) was used. The cDNA strand synthesis was carried out in Biorad S1000™ Thermal Cycler. Thermo Scientific's DyNAmo (Catalog no. F-416) reagent was used for this purpose with cDNA from good quality RNA used as template. Applied Biosystems™ 7900HT Fast Real-Time PCR system was used. GAPDH was used as internal control.

### Bisulfite sequencing of promoter region

Genomic DNA was extracted using QIAamp DNA Mini kit (Qiagen, USA). Isolated and purified genomic DNA was subjected for bisulfite conversion using EZ DNA methylation kit (Zymo Research, USA). The promoter region of GNG4 was amplified using methylation as well as unmethylation specific primers and the fragments were cloned into pGEMT-EZ vector system using TA cloning. Finally, the cloned fragments were subjected to Sanger Sequencing using M13 forward primer. Ten such clones were sequenced for each sample and the average of the methylation levels for each CpG was plotted using the lollipop diagram.

### Azacytidine treatment

Cells were plated in 35mm dishes and after 24 hours, azacytidine treatment (5 μM and 10 μM) was given and after 4 and 6 days the cells were harvested in Tri-reagent. RNA was isolated from each sample, and after cDNA conversion, real time PCR was carried out to quantify expression compared to untreated cells.

### Activation of CXCR4 by SDF1α

Before activation, cells were starved in minimal media for 12 hours to attain basal level signaling. 50 nM BSA (Sigma, Cat. No. A9418) or purified SDF1α (R&D Systems, Cat. No. 350-NS) ligand was added. For western blotting, cells were harvested after 20 minutes of addition of ligand using RIPA buffer. For scratch assay, SDF1α was replenished every 4 hours. For Boyden-chamber assay, SDF1α was replenished every 2 hours.

### Boyden-chamber assay

50,000 U87MG cells harboring VC or GNG4 constructs were plated in the upper chamber in serum-free DMEM. 50 nM of BSA or SDF1α was added in DMEM in the lower chamber. The cells were allowed to migrate for six hours after which the cells were fixed in 100% chilled methanol and stained using 0.2% Crystal violet stain.

### Pharmacological inhibitors

Erk and Jnk inhibitors used include U0126 (Calbiochem; Cat. No. CAS 109511 -58-2) and SP600125 (Sigma; Cat. No. S5567) respectively. U0126 was used at 10nM and SP600125 was used at 50nM concentration. For Boyden chamber assay, the inhibitors were added along with the cells in the upper chamber and cells were allowed to migrate for six hours.

### Analysis of phosphorylation levels of MEK, JNK and ERK from TCGA data

CXCR4 over expressing GBM samples were those which show RNA levels of CXCR4 greater than two fold in the log 2 ratio. These samples were further divided into GNG4 RNA high and low in the following manner:-

GNG4 high = Log2 ratio > −0.79
GNG4 low = Log2 ratio > −0.79

This cut-off was considered as −0.79 is the median value for RNA levels of GNG4 in TCGA GBM samples. The phospho protein values were obtained from TCGA RPPA data.

## SUPPLEMENTARY MATERIALS FIGURES




