# Supplementary material for: Epigenetically silenced GNG4 inhibits SDF1α/CXCR4 signaling in mesenchymal glioblastoma
Source: Genes Cancer. 2016 Mar;7(3-4):136–47. doi: 10.18632/genesandcancer.105 (PMC4918951; doi:10.18632/genesandcancer.105)
Supplement: Supplementary file 1 [file ganc-07-136-s001.pdf]

Epigenetically silenced GNG4 inhibits SDF1α/CXCR4 signaling in mesenchymal glioblastoma - Pal et al

A

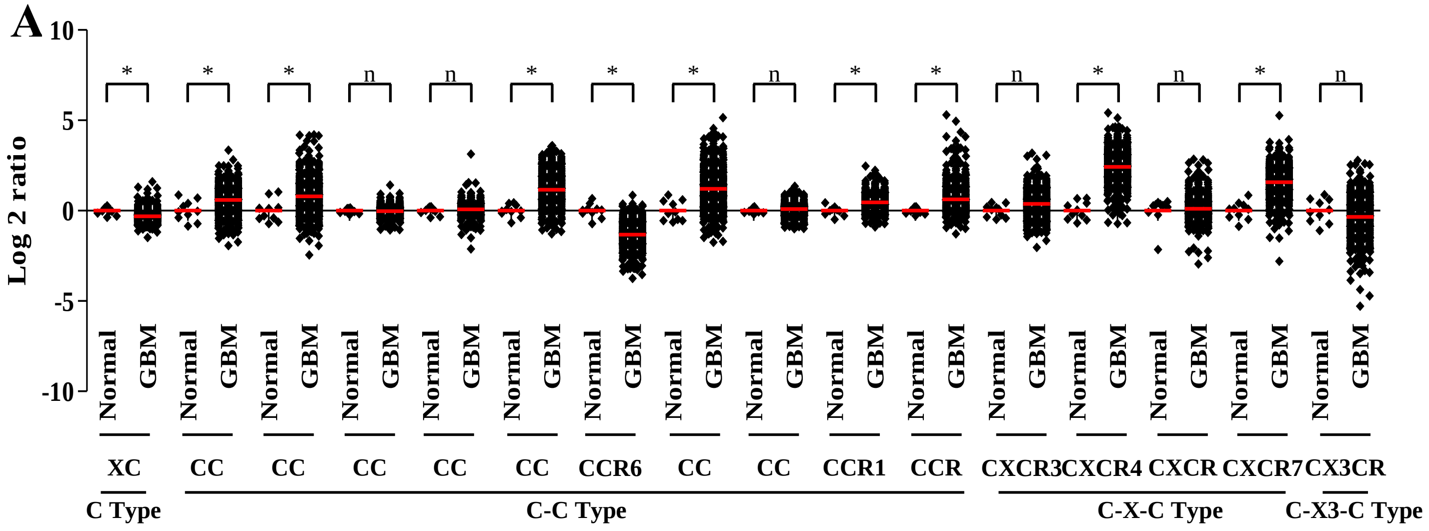

B

Family	Chemokine Receptor	TCGA Agilent	Log2 ratio	p value	Corelation with GNG4	
					Spearman r	pvalue
C Family	XCR1	Un-regulated	-0.31	0.00	0.02	0.567
C-C Family	CCR1	Up-regulated	0.60	0.01	-0.37	0.000
	CCR2	Up-regulated	0.79	0.01	-0.43	0.000
	CCR3	Un-regulated	-0.02	0.84	-0.11	0.007
	CCR4	Un-regulated	0.08	0.41	-0.11	0.007
	CCR5	Up-regulated	1.15	0.00	-0.33	0.000
	CCR6	Down-regulated	-1.32	0.00	-0.17	0.000
	CCR7	Up-regulated	1.21	0.00	-0.28	0.000
	CCR8	Un-regulated	0.09	0.10	-0.02	0.642
	CCR9	No Data				
	CCR10	Un-regulated	0.46	0.00	-0.13	0.001
	CCRL1	Up-regulated	0.62	0.00	-0.19	0.000
C-X-C Family	CXCR1/2	No Data				
	CXCR3	Un-regulated	0.37	0.06	-0.29	0.000
	CXCR4	Up-regulated	2.43	0.00	-0.42	0.000
	CXCR5	No Data				
	CXCR6	Un-regulated	0.10	0.39	-0.12	0.004
	CXCR7	Up-regulated	1.58	0.00	-0.10	0.019
C-X3-C Family	CX3CR1	Un-regulated	-0.34	0.35	-0.18	0.000

Supplementary figure 1: Regulation of different chemokine receptors in GBM. A) RNA levels of different chemokine receptors in GBM versus control brain samples, p-value was calculated by Student's t test where *, ** and *** represents p-value of < 0.05, 0.01 and 0.001 respectively, **B)** Table showing regulation of different chemokine receptors in GBM and their correlation with that of GNG4. Along with p-value significance, genes with RNA level of Log2 ratio > 0.57 was considered to be biologically significant and hence, up regulated.

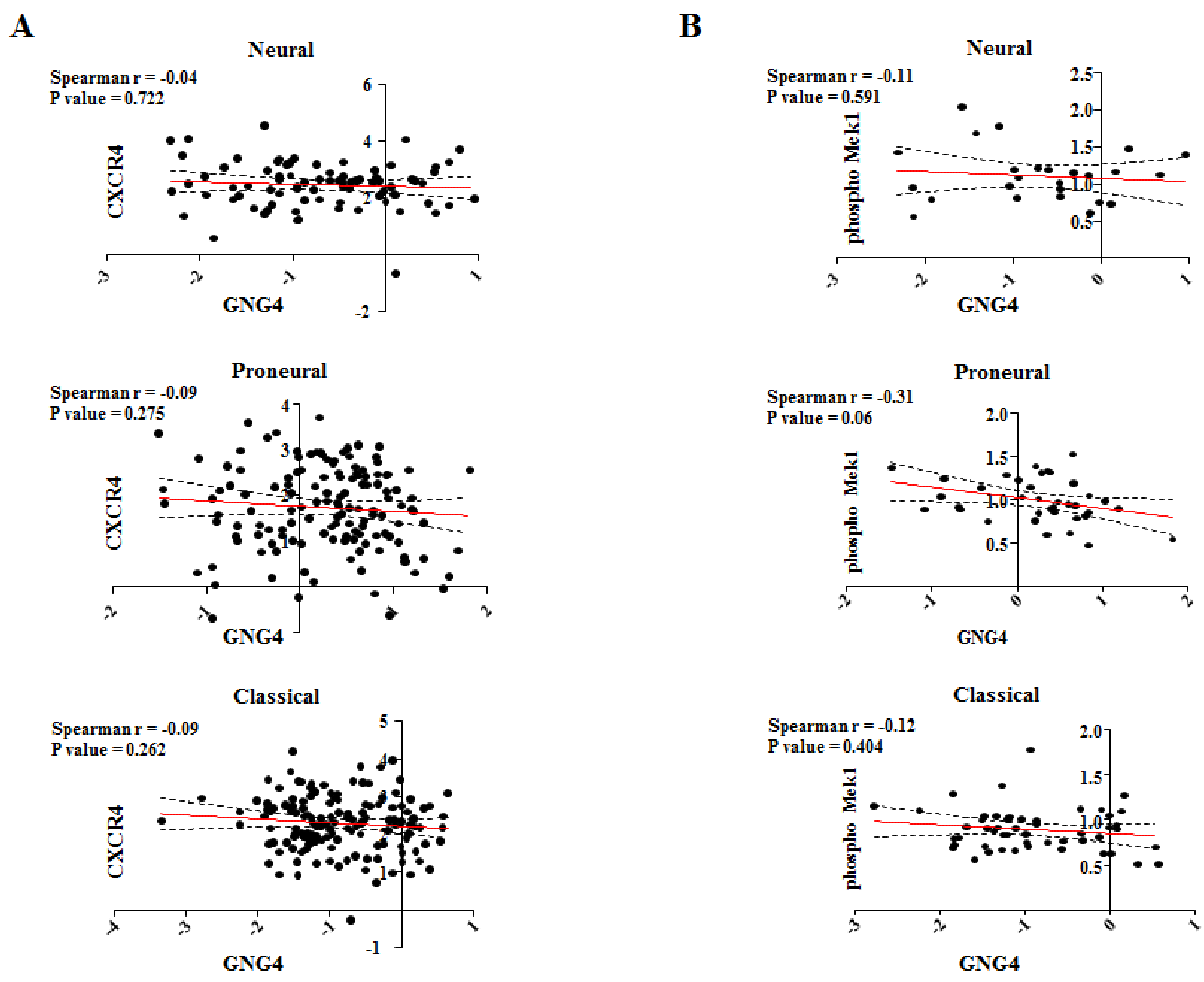

Supplementary figure 2: Correlation of GNG4 RNA levels with CXCR4 in different subtypes of GBM. A) RNA level correlation of GNG4 and CXCR4 in subtypes of GBM from TCGA Agilent microarray data. **B)** Correlation between RNA level of GNG4 and protein level of phospho-Mek1 in subtypes of GBM from TCGA data.

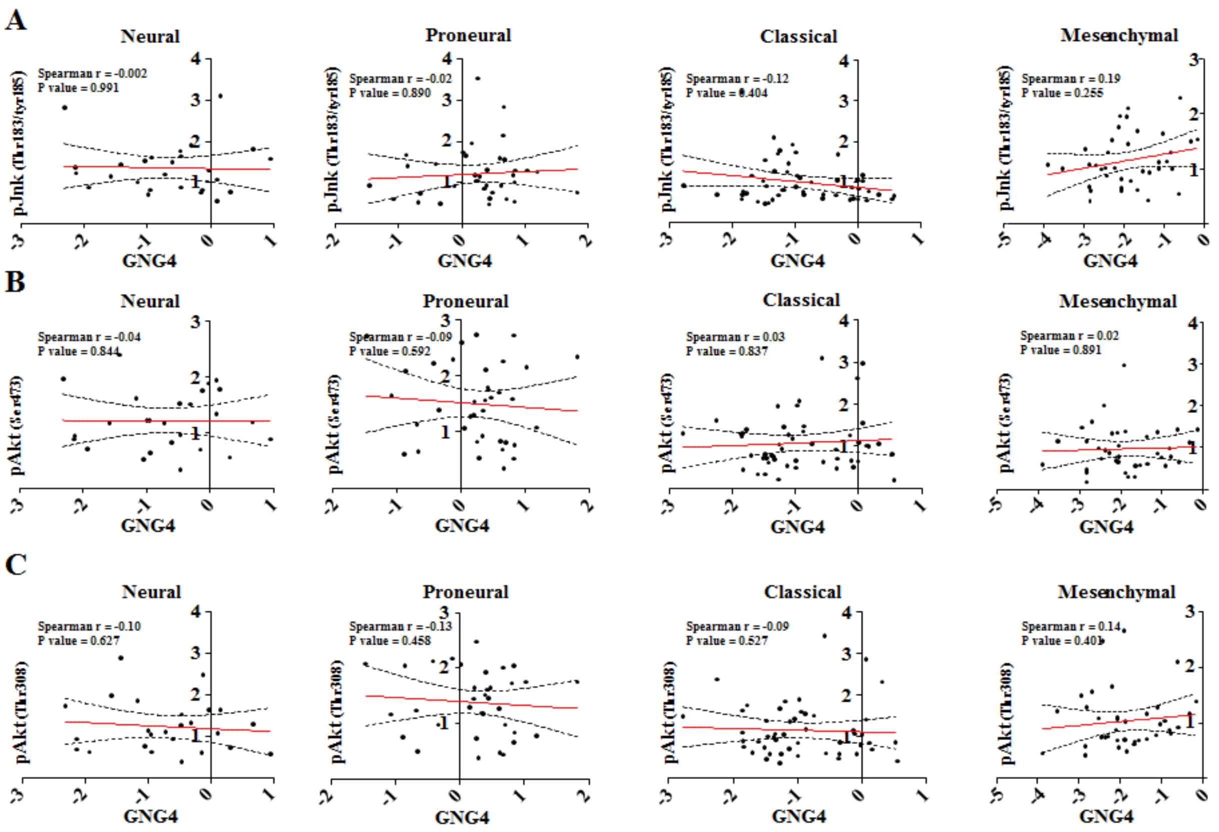

Supplementary figure 3: Correlation of GNG4 RNA levels with phospho-Jnk and phospho-Akt in different subtypes of GBM. A) RNA levels of GNG4 correlated with phospho-Jnk (Thr183/Tyr185) in different GBM subtypes from TCGA data, **A)** RNA levels of GNG4 correlated with phospho-Akt (Ser473) in different GBM subtypes from TCGA data, **A)** RNA levels of GNG4 correlated with phospho-Akt (Thr308) in different GBM subtypes from TCGA data.
